# Clinical evidence of human pathogens implicated in Alzheimer’s disease pathology and the therapeutic efficacy of antimicrobials: an overview

**DOI:** 10.1186/s40035-023-00369-7

**Published:** 2023-07-26

**Authors:** Celso S. G. Catumbela, Vijayasree V. Giridharan, Tatiana Barichello, Rodrigo Morales

**Affiliations:** 1 Department of Neurology, McGovern Medical School, The University of Texas Health Science Center at Houston, Houston, TX 77030 USA; 2 Translational Psychiatry Program, Faillace Department of Psychiatry and Behavioral Sciences, McGovern Medical School, The University of Texas Health Science Center at Houston, Houston, TX 77054 USA; 3 Graduate Program in Health Sciences, University of Southern Santa Catarina (UNESC), Criciúma, SC 88806-000 Brazil; 4 Centro Integrativo de Biologia y Quimica Aplicada (CIBQA), Universidad Bernardo O’Higgins, 8370993 Santiago, Chile

**Keywords:** Alzheimer’s disease, Amyloid-β, Pathogens, Infections

## Abstract

A wealth of pre-clinical reports and data derived from human subjects and brain autopsies suggest that microbial infections are relevant to Alzheimer’s disease (AD). This has inspired the hypothesis that microbial infections increase the risk or even trigger the onset of AD. Multiple models have been developed to explain the increase in pathogenic microbes in AD patients. Although this hypothesis is well accepted in the field, it is not yet clear whether microbial neuroinvasion is a cause of AD or a consequence of the pathological changes experienced by the demented brain. Along the same line, the gut microbiome has also been proposed as a modulator of AD. In this review, we focus on human-based evidence demonstrating the elevated abundance of microbes and microbe-derived molecules in AD hosts as well as their interactions with AD hallmarks. Further, the direct-purpose and potential off-target effects underpinning the efficacy of anti-microbial treatments in AD are also addressed.

## Background

**Fig. 1:**
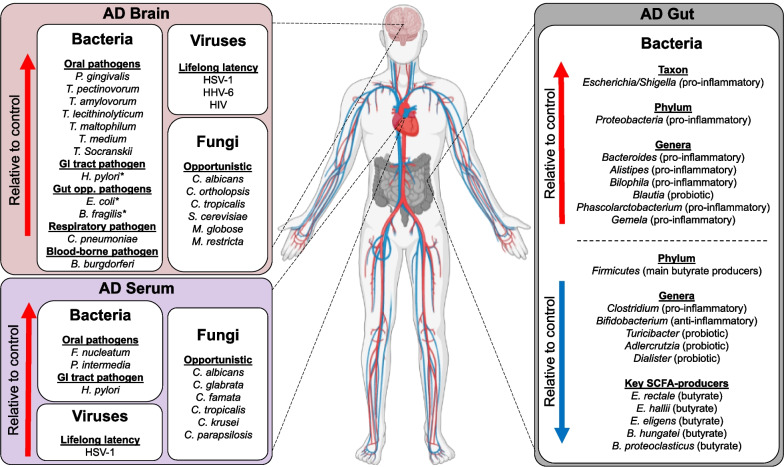
Diagram depicting major pathogens associated with Alzheimer’s disease in brain, gut and serum. Gut opp. pathogens: gut opportunistic pathogens; *indicates the cerebral presence of microbe-derived molecules only (e.g., associated LPS, toxins, and immunoglobulin G). Figure created using BioRender

More than a century ago, Aloysius Alzheimer proposed that microorganisms may be involved in the progression of dementia and associated pathological features such as the formation of senile plaques [1]. At present, the specific causes leading to Alzheimer’s disease (AD) are still unidentified, although various co-morbidities and lifestyle patterns have been linked to disease incidence [2–4]. In recent decades, several studies done in human-derived samples and experimental models have connected pathogens and associated inflammatory pathways to AD pathology [5–9]. Indeed, although microbes exhibit the ability to infiltrate the brain of cognitively healthy persons at various life stages, numerous reports indicate that the cerebral presence of microbial agents appears to be exacerbated as a consequence of AD-like pathological events, such as significant disruption of the blood–brain barrier (BBB), elevated neuroinflammation, and alarmingly, higher cerebral levels of amyloid-β (Aβ) peptide—the predominantly suspected driver of AD pathology [9–13]. Perhaps, the disruption of glial function that is characteristic of AD brains impairs the clearance of microbes from this tissue. Yet, the direction of causality remains unclear due to either conflicting results or lack of more comprehensive studies; and thus, is still a major, even controversial topic of AD research. Excellent reviews have addressed the growing experimental observations in support of an infectious etiology for AD, even discussing associated hypotheses [14–17]. Here, we evaluate the putative pathological crosstalk between pathogens and AD through a clinical lens only, as in the absence of experimental observations—which are at a greater risk of identifying non-realistic interactions between microbes and features of dementia—the underlying mechanisms can be scrutinized in different fashion compared to reviews by others. Specifically, we present data derived from analyses of the Google Scholar, PubMed, Embase, Scopus, and ClinicalTrials.gov registries outlining the clinical evidence of pathogens associated with brain and peripheral tissues from AD patients (Fig. 1). Importantly, we also include reports of the efficacy of antimicrobial therapeutics against AD to provide an additional frame of reference wherein the link between infection and dementia may be evaluated (Table 1).

**Table 1:** Peer-reviewed clinical trials associated with positive Alzheimer’s disease outcomes following treatment with antimicrobials

Antimicrobial(s)	On-target effect(s)	Off-target effect(s) on AD-associated processes	Clinical trial: Study population	Clinical trial: Treatment dosage	Clinical trial: Improved AD outcomes
Cycloserine	Broad-spectrum antibiotic [18]	Inhibits: PI3K/Akt pathway NO production Phosphorylation of LPS-induced ERK [19]	Probable AD patients (*n* = 91) Placebo-controlled Double-blind [20]	5, 15, or 50 mg 2 × daily (oral) 10-week duration [20]	Implicit memory performance of words repeated across trials [20]
			AD patients (*n* = 17) Placebo-controlled Double-blind [21]	50 or 100 mg 1 × daily 4-week duration [21]	ADAS-cog scores [21]
Doxycycline and Rifampin	Broad-spectrum antibiotic (both) [22, 23]	Enhances: PACAP receptors (Doxycycline) [24] Inhibits: Microglial inflammation (Rifampin) [25]	Probable AD and mild-to-moderate dementia patients (*n* = 101) Placebo-controlled Triple-blind [26]	200 and 300 mg (Doxycycline and Rifampin, respectively) 1 × daily (oral) 3-month duration [26]	Functional behavior at 3 months ADAS-cog score at 6 months [26]
*Helicobacter pylori* eradication regimen: Omeprazole, clarithromycin, and amoxicillin	Proton-pump inhibitor (Omeprazole) [27] Macrolide antibiotic (clarithromycin) [28] Broad-spectrum antibiotic (amoxicillin) [29]	Inhibits: TGF- β upregulation in vivo (amoxicillin) [30]	*H. pylori-*positive patients with AD (*n* = 56) Placebo-controlled Triple-blind [31]	In accordance to clinical standard practice in Europe > 2-year follow-up period [31]	MMSE CAMCOG FRSSD [31]
Antiherpetic treatment: Either acyclovir, famciclovir, ganciclovir, idoxuridine, penciclovir, tromantadine, valacyclovir, or valganciclovir	Antiviral [20]		Herpes simplex virus type 1 and/or type 2 patients (*n* = 8362, ≥ 50 years old) Placebo-controlled Triple-blind [32]	Self-administered, as needed Until onset of dementia > 10-year follow-up period [32]	Lower incidence of overall dementia (AD, vascular and other dementia) [32]
Sodium oligomannate (GV-971)	Inhibits Aβ fibrillization [33]	Inhibits: Gut dysbiosis Peripheral immune cells infiltration into the brain Neuroinflammation in animal models [33]	Probable AD patients (*n* = 818) Placebo-controlled Double-blind [34]	2 × daily (oral) 36-week duration [34]	ADAS-cog12 scores [34]
*N*-acetyl-*L*-cysteine (NAC)	Thiol antioxidant [35] Ameliorates psychiatric and neurological disorder-associated physiological processes [36, 37]	Broad-spectrum antibiotic [38–40]	Probable AD patients (*n* = 43) Placebo-controlled Double-blind [41]	50 mg/Kg/day ≤ 6-month duration [41]	Letter fluency task Wechsler Memory Scale [41]
			Moderate to late-stage AD patients (*n* = 12) Placebo-controlled [42]	600 mg of NAC 2 × daily (oral) ≤ 9-month duration [42]	Dementia Rating Scale scores [42]

*Aβ* Amyloid-beta, *AD* Alzheimer’s disease, *ADAS-cog* Alzheimer's Disease Assessment Scale-Cognitive subscale, *ADLs* Activities of Daily Living, *CAMCOG* Cambridge Cognitive Examination for the Elderly, *CNS* central nervous system, *FRSSD* Functional Rating Scale for Symptoms of Dementia, *LPS* lipopolysaccharides, *MMSE* Mini-Mental State Examination, *NO* nitric oxide, *ERK* extracellular signal-regulated kinase, *PACAP* pituitary adenylate cyclase-activating polypeptide, *PI3K/Akt* phosphoinositide 3-kinase/Akt

Although not the focus of this review, we must note that experimental efforts have largely reinvigorated the suspicion of an infectious etiology for AD, as growing reports show that Aβ production and aggregation, as well as cerebrovascular dysfunction and neurodegeneration are exacerbated in response to the presence of some pathogens and pathogen-derived molecules in vitro and in animal models of AD [7–13, 43]. Moreover, Aβ expression is associated with a protective effect against infection in a transgenic mouse model of AD (5 × FAD), in the nematode *Caenorhabditis elegans* and in vitro [11, 12]; and notably, this appears to be mediated by the oligomerization and fibrillization of various types of pathogens (*e.g.,* viruses, bacteria, and fungi) [11]. Although compelling, experimental findings have yet to clarify the direction of causality in human patients. Nevertheless, these data suggest that the current knowledge of the pathogenic profile of AD hosts is crucial to the understanding of AD pathogenesis. Perhaps, evaluation of the growing clinical evidence demonstrating direct and indirect interactions between various pathogens and the brain and peripheral tissues of dementia patients will better elucidate the role of microbes in AD onset and/or progression.

In the present review, we delineate the epidemiological and pathological data on the known pathogens associated with AD brains and peripheral tissues and fluids. Then, we discuss the current progress in AD clinical trials involving therapeutics that inhibit the virulence of these infectious agents.

## Microbial pathogenic profile of AD patients’ brains

**Fig. 2:**
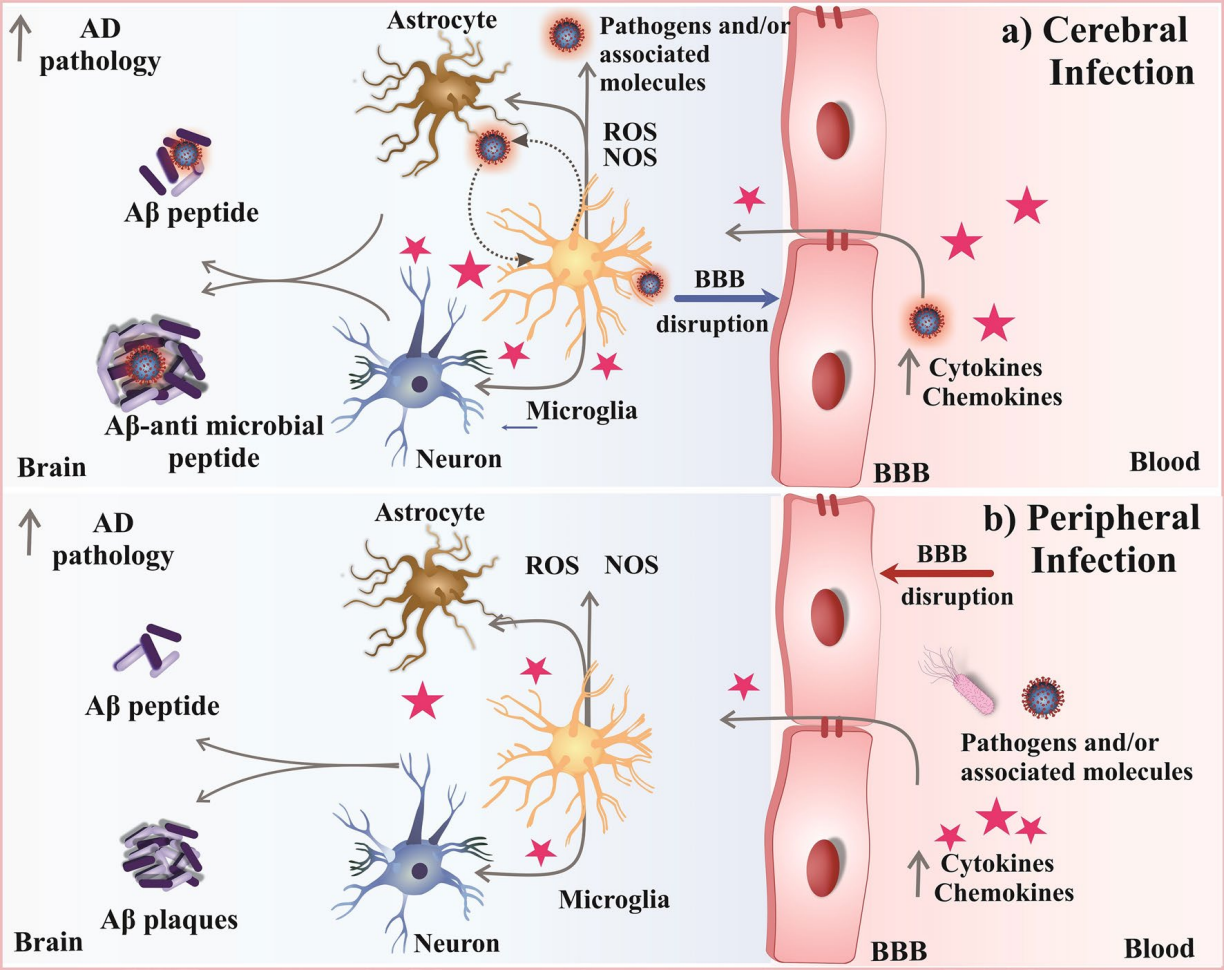
Potential mechanisms linking microbial infection with AD risks. **a** In the context of CNS infection, microglia recognize invading pathogens and engulf them. This activates inflammatory responses including the release of pro-inflammatory cytokines, increase of reactive oxygen species (ROS) and overactivation of nitric oxide synthase (NOS). Activated microglial cells lose homeostasis and produce more pro-inflammatory cytokines and chemokines, which help clear pathogens but also affect astrocytes and neuronal function. Alternatively, Aβ released from astrocytes and neuronal cells may act as an antimicrobial molecule after misfolding. However, the accumulation of misfolded Aβ species derived from this process may initiate AD pathology. **b** During peripheral infection, the excessive cytokines and chemokines present in blood infiltrate the BBB and activate microglia. Activated microglial cells lose homeostasis and produce high levels of pro-inflammatory cytokines and chemokines, activating astrocytes and damaging neurons. This cerebral inflammatory response exacerbates the deposition of Aβ in the brain parenchyma through multiple mechanisms, thus facilitating AD pathological cascades. AD: Alzheimer’s disease; Aβ: amyloid beta; BBB: blood–brain barrier; NOS: nitric oxide synthase; ROS: reactive oxygen species. Figure created using CorelDRAW

Brain infections are relatively rare, and they have a poor prognosis with a concomitant high death rate. The central nervous system (CNS) is mainly infected via haematogenic (arterial or venous route), adjacency (sinusitis or otitis), and neural pathways [44]. Infection of the CNS, by either pathogens or pathogen-derived molecules (*e.g.*, lipopolysaccharides, LPS), triggers an array of neuroinflammatory responses to promote the accumulation and activation of glial cells. These events involve cytokines, chemokines, complement and pattern-recognition receptors, as well as cellular and molecular immune factors. Although initially tasked with a neuroprotective role, the long-lasting activation of glia—as a result of either persistent infection or dysregulated innate immunity—leads to chronic and uncontrolled release of pro-inflammatory mediators as well as elevated oxidative and nitrosative stress. Consequently, these events are thought to eventually lead to cerebral Aβ accumulation and tau hyperphosphorylation [15] (Fig. 2a). Alternatively, pathogen accumulation in the periphery can lead to sustained inflammation that promotes BBB disruption and subsequently facilitates the entry of microbes and/or associated molecules into the brain (Fig. 2b). It has been suggested that the presence of infectious agents—either genuine or perceived—may trigger the accumulation of Aβ, as this peptide parallels classical antimicrobial proteins (AMPs) in several ways including antimicrobial activity against a range of pathogens [12, 43, 45]. These two putative models explaining the infectious etiology of AD are not mutually exclusive. Indeed, inflammatory pathways play central roles in the host’s response to pathogen activity [46, 47]. Several reports have revealed an array of pathogen-induced inflammatory events that are dependent on various factors, including the type of infectious agent and associated virulence hallmarks [48–52]. Regardless of the specific mechanisms linking microbial infections and AD, compelling evidence suggests that both disease-associated events synergize with deleterious consequences.

Below, we focus on data from clinical studies highlighting the diverse pathogens associated with the AD brain.

### Viruses

Herpes simplex virus-1 (HSV-1) is estimated to affect nearly 70% of the global population aged 0–49 years [53]. HSV-1 resides lifelong in the trigeminal ganglia [54]. Interestingly, studies of brains from HSV-1-positive individuals show accumulation of the pathogen in areas predominantly affected by AD, such as the hippocampus as well as the temporal and frontal cortex regions [55]. Moreover, the *APOE-*ε4 allele, a well-established risk factor for AD, modulates the host’s susceptibility to infection by numerous pathogens, including HSV-1 [56–58], which is in line with the suspicion of viral involvement in the etiology of AD.

In 1991, Jamieson and colleagues reported significantly higher proportions of HSV-1 DNA in AD brains compared to controls [59]. This finding bolstered the long-suspected connection between viruses and AD [55, 60]. Itzhaki et al*.* [61] later revealed that the *APOE*-ε4 allele frequency was significantly higher in HSV-1-positive AD brains, relative to the HSV-1-negative AD group as well as the HSV-1-positive and -negative non-AD groups. In agreement, Lin and colleagues reported significantly higher proportions of the AD brains harboring the Herpesviridae member human herpesvirus 6 (HHV6) than the normal brains; however, the authors observed no significant differences in the frequencies of HSV-2, HHV2, and cytomegalovirus (CMV) presence between AD and control brains [62]. In contrast to Lin et al., Allnutt et al., (2020) reported that the screening of three independent AD brain repositories indicated no significant association between HHV6 and AD, but agreed with prior findings of comparable frequency of presence of CMV between AD and control brains. Additionally, Allnutt et al. [63] observed no significant differences in the level of Epstein-Barr virus between AD and control brains. Together, these findings suggest that in the event of a viral etiology for AD, HSV-1 is likely the most significant contributor among Herpesviridae members. In support of a crucial role for HSV-1 in AD, Wozniak et al*.* [64] detected plaque-associated HSV-1 DNA in the frontal and temporal regions of 72% of AD brains compared to only 24% of control brains. However, a systematic review by Warren-Gash et al*.* [65], covering seven medical databases and grey literature sources as well as considering the methods of Herpesviridae measurement, reported no difference in the detection of HSV-1 in dementia brains compared to controls. Although the putative viral etiology of AD remains unclear, clinical observations reveal that queries into the link between HSV-1 and AD are confounded by the variably efficient methods of Herpesviridae measurement and detection. Moreover, these reports may be further complicated by the complex inflammatory and non-inflammatory pathways associated with chronic and acute viral infections [66]. Clinical studies have continued to explore the link between AD and other viruses associated with lifelong persistence such as human immunodeficiency virus (HIV), which is the causative agent of acquired immunodeficiency syndrome (AIDS), a condition characterized by the progressive failure of the immune system and increased susceptibility to opportunistic and primary infections [67].

The brain is a key reservoir for HIV, as this pathogen resides in microglia and astroglia to evade the immune system [68, 69]. Numerous groups have reported a significantly higher frequency of excess dementia and peripheral neuropathy in HIV-infected individuals carrying the *APOE-*ε4 allele, which is the strongest genetic risk factor for late-onset AD [70–72]. Esiri and colleagues showed the increased prevalence of amyloid deposits (in the form of argyrophilic plaques) in the cerebral cortex of frontal and temporal lobes of AIDS patients, relative to age-matched, non-HIV-infected controls. The investigators revealed that the prevalence of plaques increased with age in both the control and AIDS groups; however, relative to controls, there was a significantly greater prevalence of argyrophilic plaques in the AIDS group as a whole and in those in the fourth decade [73]. In line with this, Green et al*.* [74] found that HIV-positive patients present with elevated levels of perivascular plaques as well as the presence of 4G8- and 6E10-positive amyloid deposits predominantly in the neuronal soma and dystrophic axonal processes. Smith et al*.* [75] reported elevated tau and amyloid levels as well as increased microglial activity in HIV-positive brains compared to HIV-negative controls, and interestingly, a significant correlation of HIV proviral DNA levels in the brain with levels in the lymphoid tissue but not with those in the plasma. Together, these data suggest that HIV-infected individuals are predisposed to AD-like brain pathology.

Collectively, clinical reports indicate that viruses characterized by cerebral residence and lifelong latency (e.g., HSV-1, HHV-6, and HIV) exhibit dramatically altered activity in the AD brain. These human-derived data strongly suggest that the viral pathogens exacerbate AD pathology and gain increased access to the brain upon the onset of dementia.

### Oral bacteria

Ide and colleagues examined a cohort of mild to moderate AD patients over a six-month period and revealed that the presence or absence of periodontitis at baseline led to a significant difference in the rate of the Alzheimer’s Disease Assessment Scale-Cognitive Subscale (ADAS-cog) score change [76], implicating pathogens such as *Porphyromonas gingivalis*, a keystone periodontal bacterium, in the progression of AD. In line with this, Poole et al*.* [77] demonstrated the presence of *P. gingivalis* LPS in AD brains, but not in controls. The relevance of *P. gingivalis* to AD pathology was further expanded by Dominy and colleagues who detected both *P. gingivalis* by 16S rRNA sequencing and the *hmuY* gene (highly specific for *P. gingivalis*) in the cerebrospinal fluid (CSF) of AD patients, finding that the toxic *P. gingivalis* proteases arginine-gingipain (RgpB) and lysine-gingipain (Kgp) were significantly more frequent and abundant in the middle temporal gyrus of AD brains, relative to non-demented control tissues. Remarkably, Dominy and colleagues also revealed that RgpB and Kgp co-localized with tau tangles and amyloid plaques in the AD hippocampus [7]. These results suggest that *P. gingivalis* or some of its byproducts can access the brain in AD patients and enhance inflammatory profiles, as well as interact with AD-associated proteins. In addition to *P. gingivalis*, other oral pathogens have been implicated in AD pathology, including members of the genus *Treponema*, which are characterized by a spirochete shape and distinguished as highly neuroinvasive [78–80].

The genus *Treponema* contains both pathogenic (e.g., *T. pallidum*, *T. denticola*, *T. pectinovorum*, and *T. carateum*) and non-pathogenic (e.g., *T. azotonutricium*, *T. caldarium*, *T. primitia*, and *T. succinifaciens*) bacteria [81]. Pathogenic *Treponema* spp. are major factors in the incidence and severity of human periodontal diseases [82, 83] and have become an attractive target of AD studies due to their highly invasive nature and frequent association with AD patients [84–87]. For example, *T. pallidum* is the pathogen responsible for neurosyphilis and has been reported to cause slowly progressive dementia and AD-like cerebral features such as neurofibrillary tangles, immature and mature Aβ-amyloids, and cortical atrophy associated with argyrophilic plaques [88]. Riviere et al*.* (2002) reported the significantly higher presence and increased diversity of *Treponema* pathogen DNA in the frontal lobe cortex of AD brains, relative to non-AD donors. Subsequent detection of *Treponema* DNA in the hippocampus, trigeminal ganglia, and pons suggested the trigeminal nerve as a potential route of entry for these oral pathogens [84]. To further evaluate this timely topic, Miklossy (2011) critically analyzed studies describing an association and causal relationship between spirochetes and AD, in accordance with the established criteria of Koch and Hill. The researcher found that spirochetes were frequently observed in AD brains (> 90% of AD cases) when highly prevalent *Treponemas* (*T. pectinovorum, T. amylovorum, T. lecithinolyticum, T. maltophilum, T. medium,* and *T. socranskii*) were studied or when studies used neutral techniques to interrogate all types of spirochetes. Remarkably, the spirochete bacterium *Borrelia burgdorferi* was 13 times more frequent in AD brains than in controls [85]. Together, these data indicate that diverse types of periodontal bacterial pathogens are frequently observed in the AD brain. Residence in the oral environment—hypothesized to be an efficient route to the brain [89]—likely underlies the frequent association between AD and these microbes, indicating that chronic inflammation of the mouth disrupts the BBB to facilitate cerebral invasion. However, for non-oral bacteria, clinical reports suggest that access to the AD brain occurs via other routes [90, 91].

### Non-oral bacteria

Compelling evidence has revealed that *C. pneumoniae*, an obligate intracellular opportunistic pathogen associated with respiratory infections, exacerbates numerous inflammatory diseases (e.g., asthma, arthritis, atherosclerosis, lung cancer, and chronic obstructive pulmonary disease) [92] as well as neurological disorders (e.g., multiple sclerosis, schizophrenia, and AD) [93]. Balin et al*.* (1998) first reported the presence of *C. pneumoniae* genes in AD brains, but not in controls. Further evaluation confirmed the viability, transcriptional activity, and intracellular presence of this pathogen in pericytes, microglia, and astroglia in the hippocampus, temporal cortex, and other AD-associated brain regions in dementia patients [94]. In line with this, Gérard and colleagues detected *C. pneumoniae* genes *Cpn*1046 and *Cpn*0695 in the brains of AD patients. They cultured *C. pneumoniae* derived from AD brain homogenate, revealing them to be viable, metabolically active, and present in astrocytes, microglia, and neurons. Notably, Gérard et al. [95] reported close proximity of the pathogen to neurofibrillary tangles and neuritic senile plaques. Together, these findings indicate that *C. pneumoniae* proliferates in the AD patient brain, perhaps interacting with AD-associated pathogenic protein aggregates and glial cells tasked with mitigating disease progression.

In addition to respiratory pathogens, byproducts from bacteria endemic to other peripheral tissues have been observed in the AD patient brain. Kountouras et al. [96] identified significantly higher concentrations of anti-*Helicobacter pylori* IgG in the CSF of AD patients compared to controls. Similarly, clinical evidence for the cerebral presence of gut bacteria is still inconclusive due to the involvement of pathogen-derived molecules rather than the actual microbe. Indeed, microbe-derived molecules in the periphery may be transported to the brain in the absence of the associated microbe; and notably, these molecules are sufficiently immunogenic to induce inflammation and other pathologies including AD-related changes [97, 98]. Zhao et al*.* [99] reported that LPS specific to *Bacteroides fragilis* and *Escherichia coli* (together constituting 35%–40% of all gut bacteria) is increased in the lysates of the hippocampus and superior temporal lobe neocortex of AD brains, relative to age-matched controls. Remarkably, the authors found that some hippocampal samples from advanced AD brains showed a 26-fold increase in bacterial LPS levels. Further, Zhan and colleagues showed that the *E. coli* K99 pili protein and LPS levels were higher in AD brains compared to controls. They demonstrated that the *E. coli* LPS was present in neurons, oligodendrocytes, microglia, and oligodendrocyte progenitor cells as well as being co-localized with Aβ in amyloid plaques and around blood vessels [100]. The results suggest that beyond their crucial role as mediators of the gut-brain-axis crosstalk, and perhaps, independent of cerebral translocation of the microbe, the gastrointestinal (GI) tract commensal bacteria may contribute to AD pathology via effects on the brain parenchyma and vascular tissue.

Collectively, these data demonstrate that AD patient brains are susceptible to direct and/or indirect effects exerted by bacterial pathogens native to the oral, respiratory and GI tract.

### Fungi

Clinical evidence for the interaction between fungal infections and AD has been primarily put forth by Carrasco and colleagues; and thus, additional independent studies are needed to allow more conclusive evaluation of findings, as well as to minimize the risk of false positives that result from fungal colonization in the brain either at the terminal stages of AD or *post mortem* without any causal role. In 2016, the Carrasco group revealed the presence of fungal proteins (enolase and β-tubulin) and polysaccharides (chitin) in the lateral frontal cortex, cerebellar cortex, entorhinal cortex/hippocampus and choroid plexus sections of AD patients, whereas control brains showed negligible levels [101]. In another study, Pisa and colleagues identified fungal cells and hyphae in the external frontal cortex, cerebellar hemisphere, entorhinal cortex/hippocampus and choroid plexus of AD patients, which were absent from control individuals. Moreover, the authors also detected fungal material in the neurovascular system and several fungal pathogens (endemic to various host sites) in the CNS of AD patients, including *Candida albicans*, *C. ortholopsis, C. tropicalis*, *Sacharomyces cerevisiae*, *Malassezia globose*, and *M. restricta* [102]. Consistently, Alonso and colleagues detected fungal species in the entorhinal and frontal cortices of AD brains, which belong to common and rare opportunistic genera (e.g., *Candida, Alternaria, Botrytis,* and *Malassezia*). Remarkably, they also reported AD brains co-infected with fungi and several bacteria, which suggests that fungal infections may even facilitate or be influenced by the activity of non-fungal pathogens in AD patients [103]. Collectively, these data indicate that AD hosts are at a significant risk of cerebral infection by diverse opportunistic fungal pathogens, which can coincide with bacterial infections. In addition, these findings suggest that AD brains are susceptible to variably invasive fungi, such as those highly efficient at crossing the BBB (e.g., *C. albicans*) [104] or not (e.g., *S. cerevisiae*) [105], and ultimately reveal that BBB disruption may not be required for the migration of fungal pathogens to the brain.

In summary, viral, bacterial, and fungal infectious agents directly and/or indirectly interact with neuronal cells, affected compartments, and AD misfolded proteins (Aβ and tau). Clinical data also show that the pathogenic profile associated with AD brains is dramatically altered compared to non-demented brains. However, these reports have not determined whether the AD state facilitates the migration of pathogens from the periphery to the brain, or whether infectious agents access the brain via AD-independent mechanisms. Notably, cerebral access is not required for the onset of pathogen-induced cognitive impairments. For example, septic-associated encephalopathy (SAE) is characterized by cognitive dysfunction due to peripheral/systemic infection. SAE is estimated to occur in up to 78% of sepsis survivors [104, 105] and has long prompted the suspicion that even bloodstream pathogens contribute to AD pathology [15]. We elaborate on this in the following sections.

## Pathogenic profile of AD patient blood

Immunosenescence is the name given to the age-related decline in immune function [106]. Immunosenescence is associated with low-grade chronic inflammation leading to progressive reduction of the ability to trigger effective antibody and cellular responses against pathogens and vaccinations [107], and therefore, is a key contributor to the greater susceptibility to infections in the elderly population [108]. This is partly due to the impairment in neutrophil function, extracellular trap formation and bactericidal effect, as well as decreased cytokine production and reduction in macrophage chemotaxis and phagocytosis [109]. Other factors that leave the elderly susceptible to infection include reduced nutritional status, swallowing difficulty, decreased mucociliary clearance, and altered gut microbiome status, among others [110]. Additionally, co-morbid conditions such as type-2 diabetes, high blood pressure, and dementia, as well as long-term hospitalizations are associated with an increased risk of infection in older adults [111]. Together, these events impair the clearance of microorganisms from circulation, and as clinical data indicate, may predispose AD patients to the accumulation of distinct pathogens in the bloodstream.

### Viruses

In 1987, Renvoize et al*.* [112] reported comparable serum levels of viral pathogens such as influenza A and B, adenovirus, measles, CMV, and HSV between AD and non-demented controls. In 1990, Ounanian et al. [113]confirmed that antiviral antibody titers were not significantly altered between AD and control patients; however, they found that AD patients were more frequently associated with autoantibodies and showed an increased prevalence of antibodies to spectrin, peroxidase, and thyroglobulin. The findings of Ounanian and colleagues suggest that AD patients have increased autoimmune responses. Letenneur et al*.* [114] reported that the risk of developing AD significantly increased in HSV IgM-seropositive individuals (indicative of primary infection/reactivation of the virus), but not in IgG-seropositive counterparts (indicative of lifelong HSV infection). Consistently, Lövheim et al*.* [115] showed that the HSV IgM seropositivity nearly doubled the risk of developing AD, whereas IgG seropositivity was not associated with the risk of disease. So far, reports have not differentiated between the contributions of HSV-1 and HSV-2 to AD pathology. Nevertheless, these data suggest that HSV-induced activation of the host’s innate immune response contributes to AD pathogenesis.

### Bacteria

The established role of peripheral immunity in AD pathology [113, 114] has led to increasing interest in disseminated microbial infection in these patients. Kountouras et al*.* [115] detected increased levels of anti-*H. pylori*-specific IgG in AD patient sera compared to controls. Moreover, Stein et al*.* [116] reported that the levels of the commensal oral bacteria *Fusobacterium nucleatum* and *Prevotella intermedia* were significantly increased in AD patient serum at baseline measurement as well as post-AD diagnosis. Interestingly, Kamer et al*.* [117] demonstrated that even in cognitively normal, healthy, and aged patients, there is a positive correlation between periodontal infection/inflammation burden and uptake of the ^11^C-Pittsburgh compound B (PIB) (indicative of fibrillar Aβ plaque-binding) in brain regions such as the inferior parietal lobule, lateral temporal lobe, middle frontal gyrus, posterior cingulate cortex/precuneus, and prefrontal cortex. Together, these findings suggest that activation of the peripheral immune response due to bacterial dissemination promotes AD-associated cerebral pathology; and in AD patients, it is associated with the accumulation of oral and non-oral bacteria in the bloodstream.

### Fungi

Notably, few studies have explored disseminated fungal infections in AD patients. Therefore, at present, they should be regarded with some skepticism due to the lack of independent efforts to confirm these data. Alonso et al*.* (2014) revealed presence of high levels of fungal polysaccharides in the peripheral blood of AD patients. Further, the authors showed that AD sera contained fungal (1,3)-β-glucan as well as antibodies against an array of yeast species and fungal proteins [118]. Although considerably limited, these data demonstrated that AD patients also present disseminated fungal infections.

Collectively, queries into the pathogenic profile of AD patient blood reveal that diverse types of pathogens may contribute to the peripheral immune response of AD patients. These clinical studies also suggest that activation of the innate immune system by circulating pathogens increases the risk of developing AD, which has caused a shift of study focus to the frequent origin of bloodstream infections; namely, the gut (as discussed below).

## Pathogenic profile of AD patients’ guts

Understanding the impact of aging on the status of the gut microbiota is essential as it closely links to the host’s health. Relative to the adolescent gut, the microbiota of adults are stable and primarily influenced by diet, lifestyle, and infection [119]. Intestinal stability is crucially maintained by chemical and physical barriers (e.g., AMPs and mucus, respectively) that are modulated by the host immune cells and gut biota to mitigate infection by invading microorganisms and importantly, also act to spatially segregate the host and non-host cells to avoid unnecessary immune responses to gut commensal microbes (e.g., *E. coli*) [120]. The health of intestinal microbiota is often represented by evaluating the ratio of phylum Firmicutes compared to the phylum Bacteroidetes (F/B ratio) [121], as these are the two most dominant bacterial phyla and major factors contributing to the regulation of gut mucosal barrier function. The F/B ratio can help identify and diagnose disease states, and assess the susceptibility to or the risk of diseases. The F/B ratio reaches the peak in adulthood (10.9) and then decreases with advanced age (0.6) [122]. Notably, many disease conditions are associated with altered F/B ratios, including obesity [123], type-2 diabetes [124], and dementia [125]. In humans, it is still unclear whether these changes are a cause or a consequence of the underlying disease condition. Below, we outline the microbial populations found to be significantly altered in the AD patient gut.

### Bacteria and their metabolites

Attempts to profile the intestinal microbiota of AD patients have revealed a loss of intestinal homeostasis and a transition toward a pro-inflammatory profile [122, 123]. Vogt and colleagues reported that AD patient stools show reduced microbiome richness compared to controls. Notably, they observed increased levels of bacteria genera associated with common and rare pathogens, including *Bacteroides, Alistipes*, *Bilophila*, *Blautia*, *Phascolarctobacterium*, and *Gemela*. In addition, the authors found decreased levels of *Clostridium*, *Bifidobacterium*, *Turicibacter*, *Adlercrutzia*, and *Dialister* genera, which are known for major probiotic and anti-inflammatory roles in the gut [126]. Similarly, Cattaneo et al. [127] reported that, relative to stools from either healthy controls or cognitively impaired patients without brain amyloidosis, stools from cognitively impaired patients with brain amyloidosis showed a higher abundance of the pro-inflammatory bacteria taxon *Escherichia/Shigella* and lower abundance of the anti-inflammatory, butyrate-producer *Eubacterium rectale*. Of note, butyrate is a short-chain fatty acid (SCFA) associated with neuroprotective properties in experimental models of AD [128], Parkinson’s disease [129], spinal cord injury [130], and neuropathic pain [131]. It is possible that the enteric bacteria contribute to AD pathology via depletion of key neuroprotective SCFAs (e.g., butyrate) and/or transition to a pro-inflammatory gut profile. In line with this, Liu et al*.* [132] revealed that compared to stool samples from healthy controls, the stool samples from AD patients showed a reduced proportion of the phylum Firmicutes—that includes the main butyrate-producing bacteria in the gut—but an increased abundance of Proteobacteria, a phylum associated with major pro-inflammatory pathogenic genera (i.e., *Escherichia*, *Helicobacter*, *Salmonella*, *Yersinia*, and *Vibrio*, etc.). In agreement, Haran and colleagues reported that AD patient stools contain lower proportions of key butyrate-producing species, including *Eubacterium* (*E. eligens*, *E. hallii*, and *E. rectale*) and *Butyrivibrio* (*B. hungatei* and *B. proteoclasticus*), relative to controls [133]. Ling et al*.* (2021) also reported that the abundance of butyrate-producing genera such as *Faecalibacterium* is decreased in AD patient stools, whereas that of lactate-producing genera such as *Bifidobacterium* is increased; and interestingly, these authors found that the Mini-Mental State Examination (MMSE), the Wechsler Adult Intelligence Scale series, and the instrumental Barthel activities of daily living scores of AD patients are positively correlated to the abundance of butyrate-producers, but negatively correlated to that of lactate-producers [134].

In summary, these reports indicate that the putative contribution of gut pathogens to AD may occur via indirect effects (e.g., reduction of butyrate-mediated neuroprotection and transition towards pro-inflammatory gut taxa) and/or through migration to the brain. Notably, gut microbiota alterations are frequently associated with a higher risk of bloodstream infections [131, 132]; and therefore, in the event of an infectious etiology for AD, dementia patients who exhibit an unhealthy gut flora may be further susceptible to AD development via the above-mentioned circulating pathogens.

## Summary of the pathogenic profile of AD patients

Clinical evidence indicates that distinct pathogens (or or associated factors) accumulate in the AD brain and peripheral tissues. Microbe-induced activation of the host immune response is associated with a substantially increased risk of developing AD. Moreover, AD risk factors (e.g., *APOE* genotype) modulate the prevalence of microorganisms in dementia patients. For example, a study of an Amazonian cohort of forager-horticulturalists revealed that amongst those with high parasitic burden, the *APOE ϵ*4 carriers had better cognitive performance than non-*ϵ*4-carriers [135]. Similarly, *APOE ϵ*4 carrier status in a large rural Ghanaian population was associated with a protective effect against infection and improved fertility among women exposed to high pathogen levels [136]. Together, these data implicate a pathological crosstalk between infection and AD; and notably, further support of this link stems from findings that antimicrobial therapeutics lead to variably improved outcomes in AD patients. Next, we will discuss clinical studies that examine the therapeutic efficacy of antibiotics and antivirals against AD. Importantly, we also address the efficacy of AD therapeutics with off-target antimicrobial effects.

## Therapeutic efficacy of antibiotics and antivirals against AD

Multiple clinical trials to test the efficacy of antimicrobial therapeutics (as single agents or combinational drug therapies) in mitigating AD clinical outcomes have either been completed or are currently underway. Here, we discuss these efforts and propose putative cellular and molecular mechanisms underlying the variable therapeutic efficacies of these drugs, considering novel links between inflammatory processes and AD.

### Antiviral therapy

Tzeng et al*.* (2018) conducted a matched cohort study of newly diagnosed HSV-positive individuals (≥ 50 years old) to examine the effect of diverse antiherpetic treatments (acyclovir, famciclovir, ganciclovir, idoxuridine, penciclovir, tromantadine, valacyclovir, and valganciclovir) on the risk of dementia. The researchers reported that over a follow-up period of 10 years, HSV-positive patients taking antiherpetic medication, either alone or in combination, showed a significantly lower incidence of overall dementia (AD, vascular and other dementia) compared to HSV-positive individuals not taking the treatment [32]. As mentioned earlier, HSV-1 resides lifelong in the trigeminal ganglia and proliferates in the AD brain, and its activation of the innate immune system correlates with an increased risk of AD. Given the well-established antiherpetic properties of these drugs [137], these data support a viral etiology for AD. Potentially, the suppression of virus-induced peripheral and cerebral immune responses mitigates AD progression in the virus-afflicted individuals.

In the past decades, numerous discoveries have shed light on the cerebral and peripheral pathogens associated with increased risk of AD onset and/or progression (detailed above). These studies have emboldened the suspicion that prevention or attenuation of pathogen-associated inflammatory signaling can mitigate the risk of dementia. Schulz and colleagues performed a U.S. nationwide retrospective cohort study of aged individuals with and without prior influenza vaccination to compare the risk of incident AD between these groups. They revealed that during a 4-year follow-up period, the elderly subjects without dementia, MCI or encephalopathy, who were vaccinated at least once, are 40% less likely to develop incident AD compared to their unvaccinated counterparts [138]. The mechanism(s) underlying the putative protective effect of influenza vaccination on AD risk remain unclear. In this context, the specificity of the influenza vaccine over AD should be validated comparing other vaccination regimens. In addition, controlled experiments in animal models may help to identify the specific pathways linking influenza vaccination and AD-brain pathology.

### Antibiotic therapy

Randolph et al*.* [18] examined whether individuals with probable AD subjected to daily oral treatments of cycloserine (a broad-spectrum oral antibiotic that is used against *Mycobacterium tuberculosis*) show improved cognitive outcomes compared to the placebo group. In that study, AD patients underwent a 6-week treatment phase followed by a crossover phase that consisted of 2 weeks of cycloserine and 2 weeks of placebo prior to a 1-week washout period. The results showed that cycloserine treatment was not associated with a significant or consistent effect on cognitive function, as measured via the MMSE [139]. Similar findings were revealed by Fakouhi et al*.* (1995) following a 26-week-long, placebo-controlled study to query the therapeutic efficacy of cycloserine as a treatment for AD. Although the authors observed improvement in the verbal implicit task performance of AD patients treated with cycloserine, the incidence and severity of cognitive impairments were similar across all treatment groups, as assayed via the Cognitive Drug Research computerized test [22] and the Dementia Rating Scale, which may have resulted from the usage of low dosages of cycloserine [23]. In line with this supposition, Schwartz et al*.* (1996) conducted a double-blind, placebo-controlled trial to determine whether oral administration of varying doses of cycloserine twice daily for 10 weeks led to positive outcomes for patients with mild-to-moderate AD. They found significant improvement of implicit memory performance of words repeated across trials [20]. In support of these findings, Tsai and colleagues showed that AD patients displayed significant improvement in ADAS-Cog scores upon daily administration of cycloserine during a 4-week, double-blind, placebo-controlled study [21]. Conversely, Jones et al*.* [140] performed a meta-analysis of 2 large, multi-center, parallel-group, 6-month duration studies and reported no significant difference between cycloserine- and placebo-treated patients. They argued that the therapeutic efficacy of cycloserine against AD needs to be clarified in further studies. Additionally, even if cycloserine is confirmed to be an efficient therapeutic against AD, this protective effect may only be partly due to the associated antimicrobial properties. Indeed, cycloserine has been reported to exert diverse anti-inflammatory effects on LPS-infected RAW 264.7 macrophages, including: (1) inhibition of the phosphoinositide 3-kinase/Akt pathway; (2) production of nitric oxide; and (3) phosphorylation of LPS-induced extracellular signal-regulated kinase [19]. Perhaps, this off-target (anti-inflammatory) effect synergizes with the antibacterial activity of the drug, or not, to mitigate the contribution of bacterial accumulation and inflammation to AD clinical outcomes.

Loeb et al*.* (2004) conducted a randomized, triple-blind, placebo-controlled trial to examine whether the combined oral administration of doxycycline and rifampin (two broad-spectrum antibiotics effective against several gram-positive and gram-negative bacteria [22, 23]) mitigates clinical outcomes for patients with probable AD and mild-to-moderate dementia. They reported that, relative to the placebo group, the antibiotic-treated patients displayed significantly less dysfunctional behavior at 3 months and significantly less decline in the cognitive score at 6 months [26]. Although these positive outcomes may be due to the broad-spectrum antibacterial activity of these drugs, it is important to note that rifampin suppresses microglial activation and promotes neuron survival against inflammation [25]. Moreover, doxycycline enhances the activity of receptors of the pituitary adenylate cyclase-activating polypeptide (PACAP), which is a potent neurotrophic and neuroprotective peptide in the CNS [24]. PACAP has been reported to be significantly decreased in AD patients compared to controls [141], and its daily administration improves the cognitive outcomes of AD transgenic mouse models [142]. In addition, there is an inverse relationship between AD-dementia severity and PACAP levels in the CSF, the superior frontal gyrus, and the middle temporal gyrus of afflicted patients [141]. Potentially, these combined properties lead to the suppression of neuroinflammation and brain atrophy in AD patients, which contributes to the observed improvement in clinical outcomes.

Kountouras et al*.* [27] conducted a follow-up of *H. pylori*-positive AD patients treated with an *H. pylori* eradication regimen consisting of omeprazole (proton-pump inhibitor), clarithromycin (macrolide antibiotic also used for treatment of Lyme disease [28]) and amoxicillin (penicillin-type antibiotic [29]). They reported that at the 2-year clinical endpoint, the AD patients successfully treated with the *H. pylori* eradication regimen showed significantly improved scores of MMSE, the Cambridge Cognitive Examination for the Elderly, and the Functional Rating Scale for Symptoms of Dementia, compared to patients who failed to show reduced *H. pylori* levels after similar treatment [31]. These results indicate that *H. pylori* infection may contribute to AD pathology and that disease progression can be ameliorated upon eradication of this pathogen. Notably, in vivo studies suggest that amoxicillin inhibits the upregulation of transforming growth factor-β (TGF-β) [30], a cytokine implicated in AD pathology including Aβ accumulation [36], microglial activation [37], and neurodegeneration [143]. Although *H. pylori* eradication is linked to improved AD clinical outcomes, the off-target effects of associated regimens may contribute to the cognitive performance of *H. pylori*-treated AD patients.

### AD therapeutics with off-target antimicrobial effects

Sodium oligomannate, also referred to as GV-971, is an orally administered mixture of acidic linear oligosaccharides (ranging from dimers to decamers, with molecular weights ranging from 670 to 880 Da) derived from a marine brown alga. GV-971 penetrates the BBB through transporters such as type-1 glucose transporter and subsequently binds different subregions of Aβ to inhibit fibril formation and destabilize preformed fibrils [33]. A randomized, double-blind, placebo-controlled, multicenter phase III trial revealed that, relative to the placebo group, twice daily administration of GV-971 for 36 weeks resulted in significant improvement of the ADAS-cog12 score in patients with mild to moderate AD from 4 weeks and onwards [34]. These positive outcomes are suspected to be due to direct interaction between GV-971 and cerebral Aβ. Still, GV-971 persists in the gut, and as evidenced in animal models, promotes the reconditioning of gut microbiota, the inhibition of microbe-induced peripheral infiltration of immune cells into the brain, and the suppression of neuroinflammation [33]. As noted earlier, AD patients display increased accumulation of immunogenic gut commensal bacteria-associated molecules in the brain, as well as higher intestinal abundance of pro-inflammatory taxa deficient in neuroprotective SCFAs such as butyrate. Therefore, the efficacy of GV-971 against AD may be partly or wholly due to the modulation of the gut taxa associated with AD onset and progression.

*N*-acetyl-*L*-cysteine (NAC) is an antioxidant with bactericidal and anti-biofilm-properties thought to neutralize reactive intermediates of diverse origins [35]. Compelling evidence indicates that NAC modulates pathophysiological processes involved in various psychiatric and neurological disorders [36, 37]. In a double-blind clinical study, Adair et al*.* [41] administered either NAC (50 mg/Kg per day) or placebo to patients with probable AD and observed significant improvement in secondary measures (letter fluency task and Wechsler Memory Scale), but not primary metrics (MMSE and Activities of Daily Living Scale). In another study, Remington et al*.* (2009) showed that administration of a vitamin/nutraceutical formulation containing a high dose of NAC (600 mg) led to improved Dementia Rating Scale scores for patients with moderate to late-stage AD compared to their placebo-treated counterparts. The authors also found that the formulation treatment was associated with substantial improvement in the Neuropsychiatric Inventory as well as maintenance of performance in the Alzheimer’s Disease Cooperative Study-Activities of Daily Living for longer than 9 months [42]. Of note, the therapeutic formulation tested contained diverse agents associated with various AD-relevant effects such as the reduction of Aβ generation, tau phosphorylation, γ-secretase activity and presenilin-1 expression, as well as the ability to compensate for APOE deficiency by increasing levels of ATP, acetylcholine and glutathione. However, as the high-dose constituent of this therapeutic formulation, NAC exhibits major antimicrobial effects such as the inhibition of biofilm formation by several bacteria (e.g., *Staphylococcus epidermidis*, *Streptococcus pneumoniae*, *Pseudomonas aeruginosa*, *Stenotrophomonas maltophilia*, and* Burkholderia cepacia*) [38–40]. Because these various pathogens are well established potent inducers of inflammation [144–148], it is possible that the improved AD outcomes linked to NAC are due to the suppression of pathogen-activated peripheral immune responses.

## Summary of the therapeutic efficacy of antimicrobials against AD

Clinical studies examining the therapeutic efficacies of antimicrobials against AD have provided varied results due to a myriad of factors (i.e., inclusion criteria, cohort size, follow-up period, and drugs tested, etc.). Nevertheless, antibacterial and antiviral treatments appear to improve the clinical outcomes of mild to late-stage AD patients. Remarkably, even trials employing AD therapeutics with antimicrobial off-target effects support an infectious etiology for AD. Although not the focus of our review, the therapeutic efficacy of antimicrobials against AD has also been reported in growing experimental studies that suggest a crucial role for AMPs in AD pathology. Briefly, AMPs are associated with the inhibition of Aβ oligomerization and fibrillization (e.g., cystatins), reduction of amyloid deposition (e.g., lactoferrin), and mitigation of amyloid-induced cell toxicity (e.g., α-defensin), which may be due to the direct interaction with Aβ peptides and/or modulation of inflammation to lessen amyloidosis. Moreover, the only human cathelicidin-derived AMP LL37 is linked to a positive feedback mechanism resulting in the worsening of dementia phenotypes including elevated levels of Aβ and neurofibrillary tangles, enhanced neuronal death and brain atrophy, and enlargement of lateral ventricles as well as impairment of synaptic plasticity and cognition [149]. Recently, low levels of salivary lactoferrin have even been implicated as an early AD biomarker [150], which attests to several other topics addressed herein, including the frequent observation of oral dysbiosis in aged persons that may facilitate cerebral invasion by microbes to either trigger or exacerbate AD pathology.

## Conclusions

Clinical evidence of elevated levels and altered activity of pathogens in AD patients has bolstered the suspicion of an infectious etiology of the disease. The AD state is associated with dramatic alterations of the microbial profile in the brain, the blood, and the gut. Infectious agents are shown to directly and/or indirectly interact with AD-associated compartments as well as molecular drivers of disease such as Aβ and tau in patient brains. However, clinical efforts on this topic exhibit varied rigor and reproducibility, as well as limited knowledge of subject status before microbial infection and/or onset of AD. Thus, clinical and experimental studies remain needed to distinguish between correlation and causation.

AD therapeutics that exhibit on- and off-target antimicrobial effects are associated with cognitive improvement. Perhaps, the protective effect of antimicrobial therapeutics is due to the mitigation of the contribution of infections to the progression of AD pathology. Indeed, experimental studies have revealed significant impairment of the glymphatic system following cerebral infection (e.g., pneumococcal meningitis) [151], which could exacerbate AD progression at all stages of disease. Moreover, the prolonged activation of immune cells is a central feature of AD and induces a pro-inflammatory cascade that contributes to neuronal loss. Therefore, antimicrobial therapeutics may protect against AD by inhibiting the accumulation of pathogens and/or pathogen-derived molecules in AD patient tissue(s) that could otherwise trigger immune cell activation directly (e.g., via Toll-like receptor signaling) and/or indirectly (e.g., by promoting the accumulation of Aβ, as part of an antimicrobial response) to exacerbate AD pathology—independent of the stage of disease. Notably, the efficacy of antimicrobial therapeutics against AD may be explained by their pleiotropic off-target effects, such as the modulation of TGF-β signaling exerted by therapeutics involving major regulators of oxidative stress. Clinical reports indicate that AD patients exhibit reduced cerebral levels of the TGF-β type II receptor (TβRII). This is notable because oxidative stress has been mechanistically and chronologically linked to several aspects of AD, including mitochondrial, metabolic, metal, and cell-cycle abnormalities [152, 153]. The AD-associated neuronal damage may be due in part to the disruption of mitochondrial bioenergetics and concomitant with increased oxidative damage, which can be both the cause and the consequence of mitochondrial dysfunction. Interestingly, some infections (e.g., pneumococcal meningitis) have been linked to dyshomeostasis events such as oxidative stress and mitochondrial dysfunction in the brain [154], implicating these processes as potential mechanisms underlying the link between microbes and AD pathology and suggesting that the on- and off-target effects of antimicrobial therapeutics mitigate the contribution of infection to disease pathology in a synergistic manner.

Finally, it is important to acknowledge that overmedication (also known as polypharmacy) is a major therapeutic challenge plaguing aged persons [155, 156], which could interfere with the therapeutic efficacy of antimicrobials against AD. Indeed, repetitive and longitudinal antimicrobial regimens against AD may further exacerbate the impairment of already weakened immune systems as well as worsening gut health and inflammation and/or increasing the prevalence of drug-resistant pathogens in elderly patients. Additional clinical studies employing larger patient cohorts and longer durations of treatments are crucial for determining whether the targeting of specific microbial populations is indeed an efficient therapeutic strategy against AD, which predominantly affects individuals at late stages of life.

The exploration of an infectious etiology for AD remains a complicated subject. At present, the clinical evidence of microbes associated with AD implies a role for some infections in AD pathology, and is sufficient to inspire additional efforts to unravel the peculiar link between pathogens and the leading cause of dementia.

## Data Availability

Not applicable.

## Abbreviations

Aβ: Amyloid-beta
AD: Alzheimer’s disease
ADAS-cog: Alzheimer’s Disease Assessment Scale-Cognitive subscale
ADLs: Activities of Daily Living
AIDS: Acquire Immunodeficiency Syndrome
AMP: Antimicrobial proteins
CAMCOG: Cambridge Cognitive Examination for the Elderly
CNS: Central nervous system
CSF: Cerebrospinal fluid
ERK: Extracellular signal-regulated kinase
FRSSD: Functional Rating Scale for Symptoms of Dementia
GI: Gastrointestinal
HHV: Human herpesvirus
HIV: Human immunodeficiency virus
HSV: Herpes simplex virus
LPS: Lipopolysaccharides
MMSE: Mini-Mental State Examination
NO: Nitric oxide
PACAP: Pituitary adenylate cyclase-activating polypeptide
PI3K/Akt: Phosphoinositide 3-kinase/Akt
SAE: Septic-associated encephalopathy
SCFA: Short-chain fatty acid
